# Elevated blood pressure and risk of aortic valve disease: a cohort analysis of 5.4 million UK adults

**DOI:** 10.1093/eurheartj/ehy486

**Published:** 2018-09-12

**Authors:** Kazem Rahimi, Hamid Mohseni, Amit Kiran, Jenny Tran, Milad Nazarzadeh, Fatemeh Rahimian, Mark Woodward, Terence Dwyer, Stephen MacMahon, Catherine M Otto

**Affiliations:** 1 The George Institute for Global Health, University of Oxford, Le Gros Clark Building, South Park Road, Oxford, UK; 2 Deep Medicine, Oxford Martin School, University of Oxford, UK; 3 Oxford University Hospitals NHS Foundation Trust, Oxford, UK; 4 The Collaboration Center of Meta-analysis Research, Torbat Heydariyeh University of Medical Sciences, Torbat Heydariyeh, Iran; 5 The George Institute for Global Health, University of Sydney, Sydney, Australia; 6 Department of Epidemiology, Johns Hopkins University, Baltimore MD, USA; 7 University of Washington, Seattle, Washington, USA

**Keywords:** Blood pressure, Aortic valve disease, Cohort study, Aortic stenosis, Aortic regurgitation

## Abstract

**Aims:**

To test two related hypotheses that elevated blood pressure (BP) is a risk factor for aortic valve stenosis (AS) or regurgitation (AR).

**Methods and results:**

In this cohort study of 5.4 million UK patients with no known cardiovascular disease or aortic valve disease at baseline, we investigated the relationship between BP and risk of incident AS and AR using multivariable-adjusted Cox regression models. Over a median follow-up of 9.2 years, 20 680 patients (0.38%) were diagnosed with AS and 6440 (0.12%) patients with AR. Systolic BP (SBP) was continuously related to the risk of AS and AR with no evidence of a nadir down to 115 mmHg. Each 20 mmHg increment in SBP was associated with a 41% higher risk of AS (hazard ratio 1.41, 95% confidence interval 1.38–1.45) and a 38% higher risk of AR (1.38, 1.31–1.45). Associations were stronger in younger patients but with no strong evidence for interaction by gender or body mass index. Each 10 mmHg increment in diastolic BP was associated with a 24% higher risk of AS (1.24, 1.19–1.29) but not AR (1.04, 0.97–1.11). Each 15 mmHg increment in pulse pressure was associated with a 46% greater risk of AS (1.46, 1.42–1.50) and a 53% higher risk of AR (1.53, 1.45–1.62).

**Conclusion:**

Long-term exposure to elevated BP across its whole spectrum was associated with increased risk of AS and AR. The possible causal nature of the observed associations warrants further investigation.

## Introduction

Despite growing prevalence^1^^,^^2^ modifiable causes of aortic valve disease (stenosis or regurgitation) are not well understood, and consequently, there are no effective approaches to its prevention. Given the shared pathways between several cardiovascular conditions, there has been some interest in assessment of the effect of established cardiovascular risk factors, such as elevated blood pressure (BP), on the risk of aortic valve disease.^3–5^ Mechanistic evidence for the potential causal role of BP comes from studies showing that hypertension causes abnormally high tensile stress on aortic leaflets, which can lead to endothelial injury or disruption.^6^^,^^7^ Supporting clinical evidence for the potential role of elevated BP and risk of aortic valve disease comes from a few largely cross-sectional studies that have reported a positive association between hypertension and risk of aortic stenosis (AS)^8–10^ or aortic regurgitation (AR).^11^ However, to our knowledge, no large-scale longitudinal analysis of these associations across the complete range of typical BP levels has previously been reported.

We, therefore, aimed to investigate the relationship between BP and the risk of aortic valve disease in a large contemporary population, with the null hypotheses that systolic BP (SBP) is not associated with future risk of AS or AR.

## Methods

### Data source

We used linked electronic health records from the UK Clinical Practice Research Datalink (CPRD) from 1 January 1990 to 31 December 2015. The CPRD database contains retrospective anonymised patient data from 674 general practices in the UK.^12^ It links primary care records with discharge diagnosis from secondary care (Hospital Episode Statistics), and mortality data from national death registries (Office for National Statistics). The dataset is considered the most comprehensive longitudinal primary care database with serial collection of information relating to diagnosis, treatments, investigations and outcomes^13^ and has been validated for epidemiological research for a range of conditions, including those heavily relying on imaging tests.^12^^,^^14^ Scientific approval for this study was given by the CPRD Independent Scientific Advisory Committee (ISAC). The CPRD Group has obtained ethics approval from a National Research Ethics Service Committee for all purely observational research using anonymised data from the CPRD.

### Study population

A total of 6 613 644 patients between 30 and 90 years with at least one BP measurement were identified. Patients entered the study at the date of their earliest blood pressure measurement (baseline) and exited the study at the earliest date of transfer out of the general practice as recorded in the database, death, end of the study period, or a record of aortic valve disease (whatever came first). To reduce the risk of reverse causation, we further excluded all individuals who, at baseline, had a prior diagnosis of cardiovascular disease (414 250), AS or AR (9417), or those prescribed lipid-lowering or anti-hypertensive medication (196 451). Cardiovascular disease was defined, as previously reported^15^^,^^16^ using ICD 10 and Read codes as: myocardial infarction, ischaemic heart disease, stroke, transient ischaemic attack, heart failure, chronic kidney disease, peripheral arterial disease, atrial fibrillation, or venous thromboembolism. We further excluded individuals with extreme values of baseline SBP and diastolic BP (DBP) (outside the range of 50–300 mmHg and 50–200, respectively, 14 233 patients). Patients with less than 1 year follow-up (273 567) and those with their first BP measurement before 1990 (313 543) were also excluded.

### Outcomes and exposures

Our primary outcomes were incident reports of AS or AR, which were identified from hospital discharge reports, death registers, or primary care records, using the diagnostic codes shown in Supplementary material online, *Table S1*. We excluded diagnostic codes that clearly classified aortic valve disease as congenital, or when mixed aortic valve disease was reported with no clear indication of the dominant condition. However, we included reports of rheumatic heart disease in our main analysis due to the clinical uncertainty in distinguishing between rheumatic and other non-congenital types of aortic valve disease diagnosed at middle and old age (but excluded these in a sensitivity analysis). Previous validation studies based on electronic health records have shown that the majority of clinically recorded valve disease codes are based on echocardiographic assessments and recorded cases typically represent moderate to severe severity rating.^17–19^ However, for more direct validation of valve disease and its severity in our study, three subgroup analyses were performed (see section Statistical analyses).

We defined SBP as our primary exposure because SBP has been shown to have the strongest predictive ability among most other measures of BP for most cardiovascular outcomes.^20^^,^^21^ We further chose DBP and pulse pressure (PP) (PP = SBP_mmHg_ − DBP_mmHg_) as alternative exposure variables to investigate any differential associations between BP indices on risk and to enable comparison of our findings with epidemiological studies of BP associations with other outcomes. Blood pressure measurements were taken from recordings in primary care records (not hospitals). To take account of measurement errors resulting from variability in BP measurements over longer time, we used multiple repeated BP measurements (mean of 6.7 measurements per patient) to calculate ‘regression dilution-corrected’ or ‘usual’ BP values.^15^^,^^16^^,^^22^^,^^23^ SBP, DBP, and PP were analysed as continuous variables with 20 mmHg, 10 mmHg, and 15 mmHg increments, respectively. Systolic BP was further analysed as a categorical variable: ≤120 mmHg; 121 mmHg to 140 mmHg; 141 mmHg to 160 mmHg; and ≥160 mmHg.

### Statistical analysis

We determined the association between SBP (and other BP indices) and each outcome using multivariable Cox regression and displayed hazard ratios (HRs) with floating absolute risks.^24^ We justified the proportional hazards assumption of Cox models using log cumulative hazards plots. Our primary analyses were adjusted for baseline sex, age, body mass index (BMI, kg/m^2^), smoking status (not current, no, current), cholesterol (total, LDL, HDL),^25^ year of the initial BP measurement as a categorical variable (1990 to 1994, 1995 to 1999, 2000 to 2004, 2005 to 2009, and 2010 to 2013) to control for potential period effects, and stratified for practice-level index of multiple deprivation (IMD) fifths. For BMI, smoking and lipids, we utilized the most recent measurement within 2 years of the baseline SBP measurement. If no measurement was available within this timeframe, we classified the covariate as missing. We performed subgroup analyses and report interactions by age, sex and BMI groups. We imputed missing baseline data using multiple imputation by expectation-maximization (EM) algorithm with bootstrapping,^26^ generating five imputations.^27^

To investigate the validity of our outcome variables and potential effect of valve severity on outcomes, we performed the following three sensitivity analyses. First, we extracted information on echocardiograms within 14 days prior to the recorded diagnosis of valve disease and stratified analyses by echocardiographically supported diagnoses of aortic valve disease vs. other reports with no recorded information on diagnostic tools during this 14-day interval. Second, we stratified our analyses according to whether or not there was a definitive report of valve replacement to assess whether observations might have been diluted due to inclusion of mild or asymptomatic cases. Third, we stratified our analyses by initial source of case reporting (hospital discharge vs. primary care), assuming hospital diagnoses cases to be more severe and more likely to be verified by specialists. In addition, we performed the following analyses to test the validity of our findings. First, to test the validity of methods for measuring and modelling exposure variables and co-variates, as well as the possibility of informed presence bias (i.e. higher BP leading to more investigations and detection of valve and other cardiovascular disease), we chose incident stroke as a positive control outcome and report BP associations with stroke. Second, we excluded total cholesterol, LDL and HDL as covariates from the primary analyses due to their high proportion of missing values. Third, we assessed the impact of progressive covariate adjustment to assess the robustness of our estimates to further adjustments.

Statistical significance threshold was defined as a two-sided *P*-value <0.05. Study findings are reported in accordance with the REporting of studies Conducted using Observational Routinely-collected health Data (RECORD) recommendations.^28^ Statistical analyses were performed using R, version 3.3 (R Foundation for Statistical Computing, Vienna, Austria).

## Results

In total, 5 392 183 individuals with a median follow-up of 9.2 [interquartile interval (IQI) 4.4–16] years and median age of 39 (IQI 32–53) years were included in the study. Of these individuals, 27 977 (0.52%) had a diagnosis of aortic valve disease (AS or AR). A total of 20 680 (0.38%) were diagnosed with only AS, and 6440 (0.12%) were diagnosed with only AR and were included in the analyses. The mean (standard deviation) age at the time of diagnosis were 64.2 (12.1) years and 57.0 (16.5) years, for AS and AR, respectively. Patient characteristics by SBP categories are shown in *Table 1*. Overall, 1.1% of patients had diabetes at baseline and about a third were smokers. The incidence of several vascular conditions during follow-up was higher among those with higher SBP.

**Table 1:** Baseline characteristics and incidence of aortic stenosis and regurgitation by categories of systolic blood pressure.

	<121 mmHg (*n* = 1 326 465)	121–140 mmHg (*n* = 3 553 759)	141–160 mmHg (*n* = 475 730)	>160 mmHg (*n* = 36 229)	Total (*n* = 5 392 183)
Age categories (years), *n* (%)					
30–50	1 231 610 (93)	2 641 403 (74)	129 994 (27)	4377 (12)	4 007 384 (74)
51–60	63 179 (4.8)	463 341 (13)	109 000 (23)	6996 (19)	642 516 (12)
61–70	20 764 (1.6)	272 725 (7.7)	117 556 (25)	10 372 (29)	421 417 (7.8)
71–90	10 912 (0.82)	176 290 (5)	119 180 (25)	14 484 (40)	320 866 (6)
Age (years), median (IQI)	35 (31–40)	39 (32–48)	60 (49–70)	67 (57–74)	39 (32–53)
Sex, *n* (%)						
Female	942 632 (71)	1 754 030 (49)	236 321 (50)	21 286 (59)	2 954 269 (55)
BMI (kg/m^2^) categories, *n* (%)					
less than or equal to 25	743 391 (72)	1 338 457 (50)	108 771 (33)	7725 (32)	2 198 344 (54)
26–30	221 696 (21)	910 558 (34)	131 931 (40)	9325 (39)	1 273 510 (31)
31–35	51 160 (4.9)	314 334 (12)	61 539 (18)	4567 (19)	431 600 (11)
Above 35	17 331 (1.7)	135 818 (5)	31 298 (9.4)	2451 (10)	186 898 (4.6)
Missing, %	22	24	30	34	24
BMI, median (IQI)	23 (21–26)	25 (23–28)	27 (24–31)	27 (24–31)	25 (22–28)
Smoking history, *n* (%)					
Never smoked	679 932 (58)	1 754 396 (57)	218 907 (57)	16 593 (58)	2 669 828 (57)
Ex-smoker	119 791 (10)	409 765 (13)	68 962 (18)	5362 (19)	603 880 (13)
Current smoker	365 179 (31)	912 891 (30)	94 363 (25)	6562 (23)	1 378 995 (30)
Missing, %	12	13	20	21	14
Cholesterol (mmol/L), median (IQI)					
Total	1.93 (1.66–2.24)	2.08 (1.78–6.1)	5.6 (4.8–6.4)	5.6 (4.8–6.4)	5.3 (4.6–6.1)
Missing, %	92	85	70	63	85
LDL	3.0 (2.4–3.6)	3.3 (2.6–3.9)	3.3 (2.6–4)	3.3 (2.6–4.0)	3.2 (2.6–.9)
Missing, %	96	92	84	81	92
HDL	1.4 (1.1–1.7)	1.3 (1.1–1.6)	1.3 (1.1–1.6)	1.4 (1.1–1.7)	1.3 (1.1–1.6)
Missing, %	95	90	80	77	91
Index of multiple deprivation, *n* (%)					
1st quantile	232 801 (18)	616 803 (17)	78 317 (16)	5666 (16)	933 587 (17)
2nd quantile	243 571 (18)	684 035 (19)	88 267 (19)	6429 (18)	1 022 302 (19)
3rd quantile	270 650 (20)	732 176 (21)	101 059 (21)	7712 (21)	1 111 597 (21)
4th quantile	296 419 (22)	774 452 (22)	106 427 (22)	8204 (23)	1 185 502 (22)
5th quantile	250 027 (19)	657 743 (19)	91 910 (19)	7588 (21)	1 007 268 (19)
Incidence of aortic valve disease, *n* (%)					
Aortic stenosis	931 (0.07)	12 002 (0.34)	6906 (1.5)	841 (2.3)	20 680 (0.38)
Aortic regurgitation	584 (0.044)	4104 (0.12)	1584 (0.33)	168 (0.46)	6440 (0.12)
Follow-up duration (years), median (IQI)	8.6 (3.9–15)	9.2 (4.4, 16)	11 (6.2–16)	10 (6.3–15)	9.2 (4.4, 16)
Time from first record registration to baseline (years), median (IQI)	0.049 (0–3.7)	0.12 (0.0027–7.5)	2.7 (0.016–15)	6.2 (0.038–21)	0.11 (0.0027–7.3)

IQI denotes interquartile interval; usual systolic blood pressure is derived from serial BP measurements within the median follow-up to correct for regression dilution; index of multiple deprivation is composite measure of relative deprivation at a small area level in the UK, covering an average population of 1500 people, ranked in ascending order of deprivation score and grouped in equal fifths, with quintiles 1 and 5 representing the least and most deprived areas, respectively.

**Figure 1:**
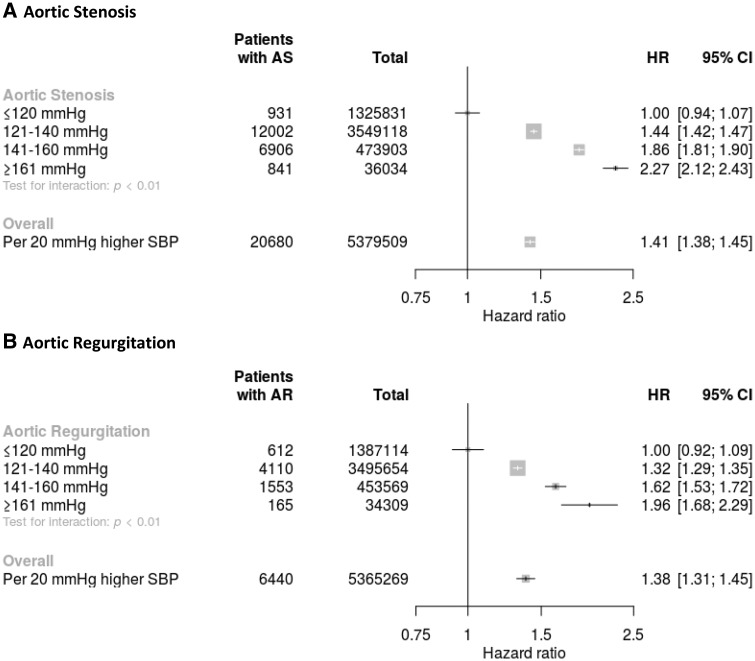
Hazard ratios for aortic stenosis (*A*) and aortic regurgitation (*B*) by categories of systolic blood pressure. Hazard ratios (HR) and 95% confidence intervals (CI) are displayed using floating absolute risk. Square sizes are inversely proportional to standard error and horizontal lines depict 95% confidence intervals. Models are adjusted for age, sex, body mass index, smoking, year of initial blood pressure measurement, total cholesterol, LDL, HDL, and practice-level index of multiple deprivation. AS, aortic stenosis; AR, aortic regurgitation; SBP, systolic blood pressure.

When SBP was analysed as a continuous variable across all age groups, each 20 mmHg increment in SBP was associated with a 1.41 times higher risk of AS (HR 1.41, confidence interval (CI) 1.38–1.45) (*Figure 1A*), and a 1.38 times higher risk of AR (HR 1.38, CI 1.31–1.45) (*Figure 1B*) with no evidence of a threshold below or above which the associations were different. Compared with the reference category of SBP ≤120 mmHg, patients with SBP ≥161 mmHg were more than twice as likely to be diagnosed with AS (HR 2.27, CI 2.12–2.43) and about twice more likely to be diagnosed with AR (HR 1.96, CI 1.68–2.29).

**Figure 2:**
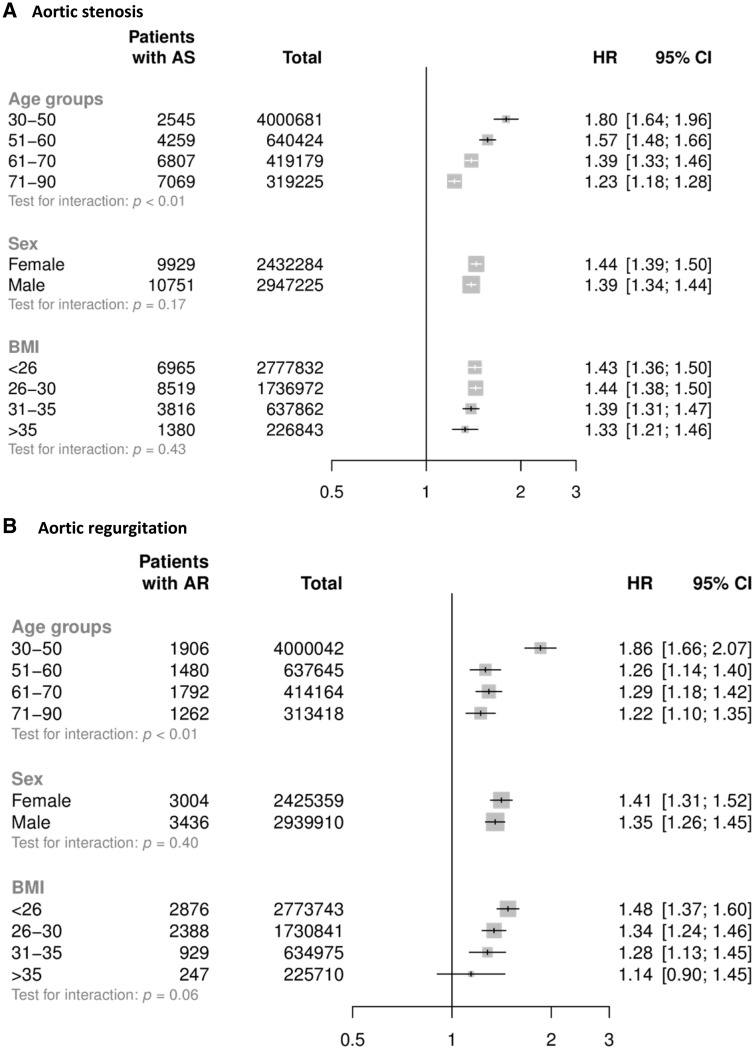
Hazard ratios for aortic stenosis (*A*) and aortic regurgitation (*B*) per 20 mmHg higher systolic blood pressure, by subgroups. Models are adjusted for age, sex, body mass index, smoking, year of initial blood pressure measurement, total cholesterol, LDL, HDL, and practice-level index of multiple deprivation. AS, aortic stenosis; AR, aortic regurgitation; BMI, body mass index.

Adjusted hazard ratios among subgroups are shown in *Figure 2*. For patients aged less than 50 years, each 20 mmHg increment in SBP was associated with a 1.80 times higher risk of AS (HR 1.80, CI 1.64–1.96); whereas in patients aged 71–90 the risk was 1.23 times higher (HR 1.23, CI 1.18–1.28) (*Figure 2A*). The corresponding hazard ratios for AR were 1.86 (CI 1.66–2.07) in the youngest age group and 1.22 (CI 1.10–1.35) in the oldest age group (*Figure 2B*). Proportional differences did not differ between men and women or by BMI categories for both AS and AR (*Figure 2*).

**Figure 3:**
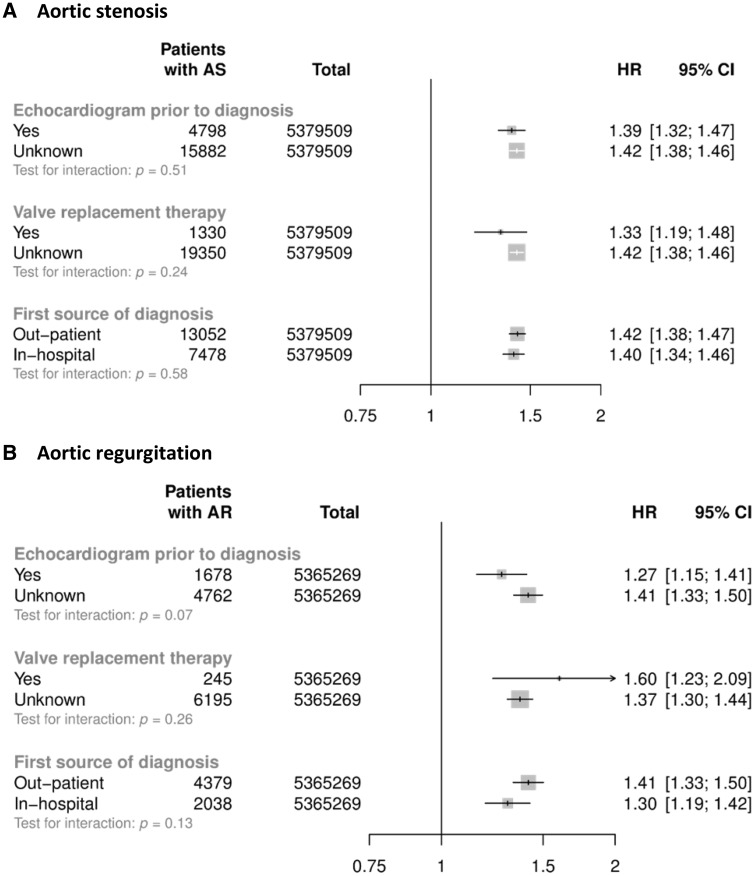
Stratified analyses for aortic stenosis (*A*) and aortic regurgitation (*B*) per 20 mmHg. Models are adjusted for sex, body mass index, smoking, calendar year of inclusion into the cohort, total cholesterol, LDL cholesterol, and HDL cholesterol.

Associations were similar when echocardiographically-supported reports were compared with reports without information on imaging (*P* for heterogeneity 0.51 for AS and 0.07 for AR); when hospital discharge reports were compared with outpatient diagnoses (*P* for heterogeneity 0.58 for AS and 0.13 for AR); or when patients with a definitive report of valve replacement were compared with those with no clear reports of valve replacement (*P* for heterogeneity 0.24 for AS and 0.26 for AR) (*Figure 3*).

**Figure 4:**
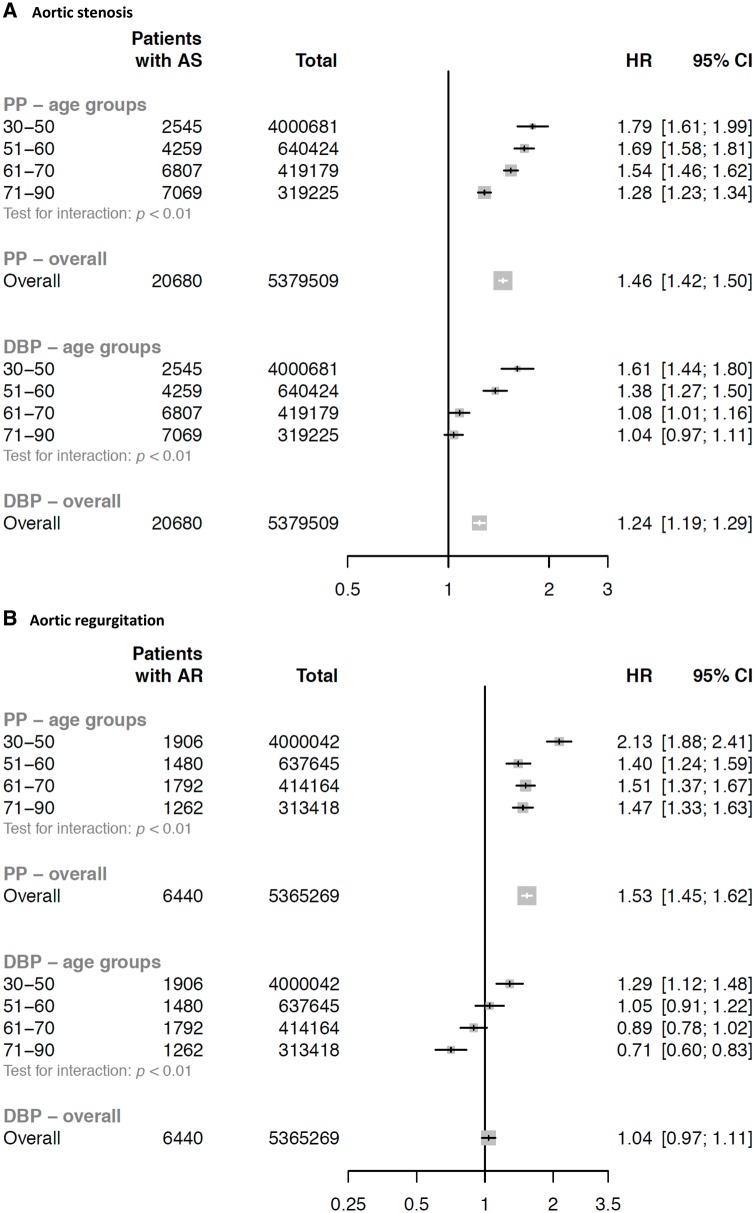
Hazard ratios for aortic stenosis (*A*) and aortic regurgitation (*B*) per 10 mmHg higher diastolic blood pressure or per 15 mmHg higher pulse pressure, by age categories. Models are adjusted for age, sex, body mass index, smoking, year of initial BP measurement, total cholesterol, LDL, HDL, and practice-level index of multiple deprivation. AS, aortic stenosis; AR, aortic regurgitation; DBP, diastolic blood pressure; PP, pulse pressure.

*Figure 4* shows the associations of PP and DBP with aortic valve disease. Each 15 mmHg higher PP was associated with a 1.46 times greater risk of AS (HR 1.46, CI 1.42–1.50), and a 1.53 times higher risk of AR (HR 1.53, CI 1.45–1.62). Each 10 mmHg higher DBP was associated with a 1.24 times greater risk of AS (HR 1.24, CI 1.19–1.29) but not significantly with AR (HR 1.04, CI 0.97–1.11). However, associations differed by age group (*Figure 4*). In addition, when PP and SBP were simultaneously entered into the models, the overall HR for SBP was substantially attenuated for AS (HR 1.08, CI 1.05–1.10) and was no longer significant for AR (HR 0.98, CI 0.94–1.03). In these SBP adjusted models, for a given SBP, DBP was negatively associated with AR or AS (Supplementary material online, *Figure S1*).

The results of additional sensitivity analyses are reported in Supplementary material online, *Appendix*. In brief, the comparison of observed associations with stroke as a positive control outcome showed no evidence of bias towards extreme: overall each 20 mmHg increment in SBP was associated with a 1.34 times higher risk of stroke (HR 1.34, CI 1.33–1.35) across all age groups. Removal of cholesterol from the adjusted models had no material effect on the finding either and progressive adjustment for covariates showed that after adjustment for age, additional adjustments for covariates had little impact on estimates.

## Discussion

In a population of adults, there was a significant association between higher SBP and increased risk of AS and AR. Proportional increases in risk for a given difference in SBP were similar throughout the range of SBP with no evidence of a threshold below or above which the relationship changed. Overall, a 20 mmHg higher SBP was associated with an approximately 40% higher risk of AS or AR, with stronger associations observed in younger groups.

A few cross-sectional studies have previously reported that patients with hypertension also have a higher chance of concomitant AS.^8–10^ One cohort study with 132 cases of AS found a positive association between systolic BP and AS.^29^ In one randomised trial of 100 patients with moderate or severe asymptomatic AS, those treated with Ramipril have a slower rate of AS progression than those allocated placebo.^30^ However, no large-scale randomised trial for prevention of aortic valve disease with BP lowering treatment has been reported. There are fewer reports of association of BP with AR, but in one cross-sectional study a positive association was seen.^11^ Consequently, current guidelines for management of valvular heart disease make no reference to strategies for prevention of AS or AR.^31^ Similarly, hypertension guidelines do not report aortic valve disease as a potential target for BP lowering treatment.^32^

To our knowledge, our study is the first longitudinal study with enough power to reliably report on age-specific associations across the whole spectrum of typical BP levels for both AS and AR. The continuous log-linear relationships between SBP and risk of aortic valve disease observed in our study are consistent with other large-scale epidemiological studies that have examined associations with other vascular outcomes.^20^^,^^21^^,^^33^^,^^34^ These findings collectively suggest that AS and AR might be partially preventable with potential implications on clinical practice guidelines for prevention of cardiovascular disease in general and valvular heart disease and hypertension in particular.^31^^,^^32^^,^^35^

Previous clinical and experimental studies have suggested that mechanical stress related to high BP plays an important role in initiating the process of aortic valve sclerosis.^36^ Higher BP and pulse pressure are associated with turbulent blood flow and shear stress.^9^ These mechanical forces contribute to endothelial cell damage and changes in the extracellular matrix, which over time lead to loss of distensibility of aorta and stiffening of the aortic valve. The ensuing progressive leaflet and aortic wall changes, in turn, contribute to left ventricular hypertrophy and further increase in SBP and PP, thus accelerating the vicious cycle of elevated BP, left ventricular hypertrophy, arteriosclerosis and further structural and functional valve damage.^36–38^

Whilst these interdependent processes have been mainly postulated to explain the pathophysiology of AS, it is not entirely clear why the same exposure, namely elevated BP, is more likely to lead to AR in some patients and to AS in others. The age-specific associations in the present study show that compared with AS, the *absolute* risk of AR increased less strongly with age. Thus, one could speculate that younger patients, who develop AR, have a higher predisposition for aortopathy or a connective tissue disorder trait, and hence respond to elevated BP and PP faster, leading to aortic root dilatation and then AR. In contrast, people without such traits may predominantly respond with arterial stiffening and aortic valve calcification over several years that eventually manifests as AS. Future mechanistic studies could explore the differences in BP pathways on AS and AR.

As an observational study, we are unable to entirely rule out uncontrolled confounding or reverse causality. Indeed, given the potential bidirectional relationship between aortic stiffness and elevated BP,^37^ it is possible that elevated SBP and pulse pressure are markers of an underlying arterial stiffness, which was not measured in our study. Ongoing Mendelian randomization studies (http://www.ukbiobank.ac.uk/2017/02/causes-and-consequences-of-valvular-heart-disease/) that are less prone to reverse causality and confounding are ideally suited to investigate the causal relationship of the observed associations in this study. A further limitation of our study is that our analyses were based on routinely collected data from linked electronic health records, which might be more prone to measurement errors of exposure and outcome variables. However, several steps were undertaken to reduce the effect of such possible measurement errors (e.g. regression dilution correction for our exposure variable) and to validate our findings (e.g. stratification of aortic valve disease cases by confirmed reports of echocardiograms or valve replacement and their comparison with stroke as a positive control outcome). Finally, studies based on clinically reported events are more likely to only capture functionally relevant and symptomatic disease states and are likely to underestimate rates compared to epidemiological studies that screen the entire population for subclinical and clinical disease. Thus, the observed associations may not be generalizable to less severe and subclinical cases of aortic valve disease.

## Conclusion

In conclusion, we found significant associations between higher BP and pulse pressure and increased risk of AS and AR. These findings suggest that risk of aortic valve disease might be modifiable but further research, ideally from meta-analyses of BP lowering trials or Mendelian randomization studies are needed to assess the causal nature of the observed associations.

## Funding

This project was supported by the ‘Oxford Martin School grant for the Deep Medicine programme’. K.R., H.M., and F.R. are supported by the ‘National Institute of Health Research (NIHR) Oxford Biomedical Research Centre’. J.T. is funded by ‘The Rhodes Trust’ and ‘The Clarendon Council’. M.W. is supported by a Principal Research Fellowship from the ‘Australian Health and Medical Research Council’.

**Conflict of interest:** none declared.

## Supplementary Material

Supplementary Data
