# Supplementary material for: Elevated blood pressure and risk of aortic valve disease: a cohort analysis of 5.4 million UK adults
Source: Eur Heart J. 2018 Sep 12;39(39):3596–603. doi: 10.1093/eurheartj/ehy486 (PMC6186276; doi:10.1093/eurheartj/ehy486)
Supplement: Supplementary Data [file ehy486_supp_data.docx]

**Elevated Blood Pressure and Risk of Aortic Valve Disease**

Kazem Rahimi FRCP, Hamid Mohseni PhD, Amit Kiran PhD, Jenny Tran MD, Milad Nazarzadeh MSc, Fatemeh Rahimian PhD, Mark Woodward PhD, Terence Dwyer MD, Stephen MacMahon FMedSci, Catherine M Otto MD

**Results 1 appendix.** Findings from supplementary analyses and their interpretation

The supplementary analyses broadly supported our main findings, which showed the following associations per each 20 mmHg SBP difference (AS: 1.38, CI 1.31, 1.45; AR: 1.41, CI 1.38, 1.45).

**1. Positive control outcome with stroke:** Supporting our main findings, this analysis did not show any evidence of bias towards extreme: overall each 20 mmHg increment in SBP was associated with a 34% higher risk of stroke (HR 1.34, CI 1.33, 1.35) across all age groups, or a 43% higher risk of stroke (HR 1.43, CI 1.40, 1.47) among the patients aged between 50 and 60 years old.

**2. Removal of total cholesterol, LDL and HDL from the adjusted models:** HRs were: AS 1.41, CI 1.38, 1.45, AR 1.37, CI 1.31, 1.45.

**3. Progressive adjustment for covariates:** This showed that after adjustment for age, additional adjustments for covariates had little impact on estimates **(Figure 2 appendix**).

## Figure 1 appendix. Hazard ratios for aortic valve disease per 10 mmHg higher diastolic blood pressure or per 15 mmHg increase in pulse pressure, by age categories.

Square sizes are inversely proportional to standard error and horizontal lines depict 95% confidence intervals. Models are adjusted for systolic blood pressure, in addition to age, gender, BMI, smoking, year of initial BP measurement, total cholesterol, LDL, HDL and practice-level index of multiple deprivation, and year of baseline BP measurement. AS = aortic stenosis; AR = aortic regurgitation; DBP = diastolic blood pressure; PP = pulse pressure

**a) Aortic stenosis**


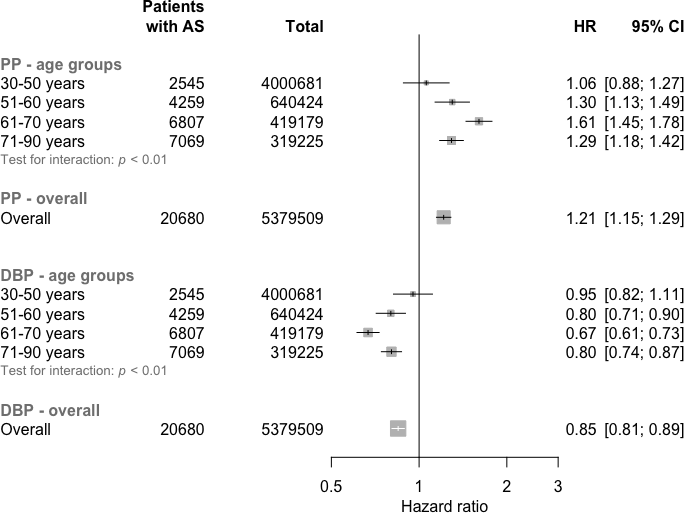


**b) Aortic regurgitation**


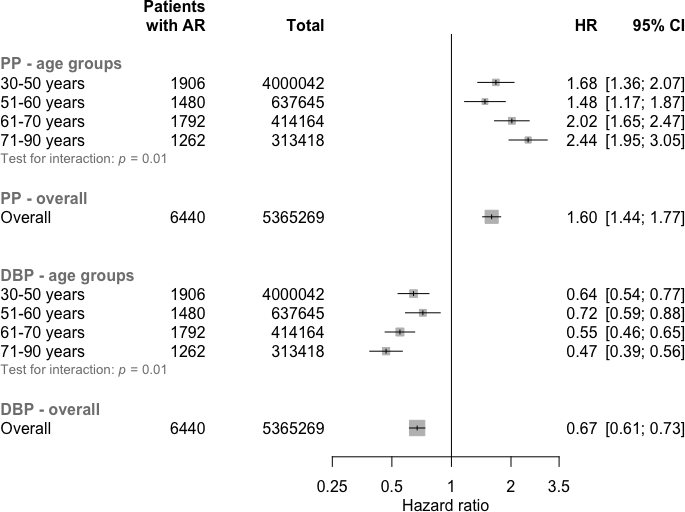


## Figure 2 appendix. Hazard ratios per 20 mmHg higher systolic blood pressure for aortic valve disease, with progressive adjustment for age, sex, calendar year of initial BP measurement, BMI, smoking and baseline LDL, HDL and cholesterol.

Square sizes are inversely proportional to standard error and horizontal lines depict 95% confidence intervals.

**a) Aortic stenosis**


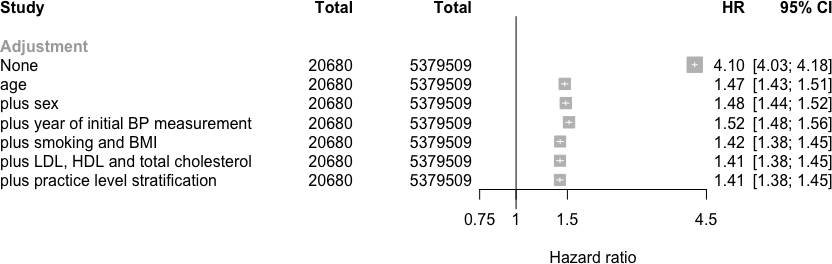


**b) Aortic regurgitation**

**
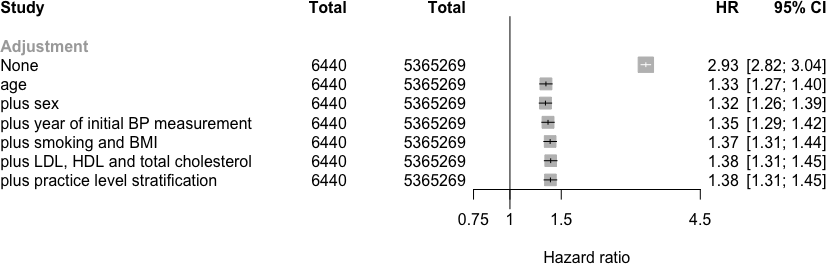
**

## Table 1 appendix. Read and ICD-10 codes for aortic valve disease.

| **Read/ICD10** | **Description** | **Stenosis** | **Regurgitation** | **Replacement** |
| --- | --- | --- | --- | --- |
| 7911100 | Xenograft replacement of aortic valve |  |  | 1 |
| 7911600 | Transluminal aortic valve implantation |  |  | 1 |
| 7911500 | Transapical aortic valve implantation |  |  | 1 |
| G122.00 | Rheumatic aortic stenosis with insufficiency | 1 |  |  |
| G120.00 | Rheumatic aortic stenosis | 1 |  |  |
| G121.00 | Rheumatic aortic insufficiency |  | 1 |  |
| 7911300 | Replacement of aortic valve NEC |  |  | 1 |
| 7911.12 | Replacement of aortic valve |  |  | 1 |
| 7911200 | Prosthetic replacement of aortic valve |  |  | 1 |
| 7911z00 | Plastic repair of aortic valve NOS |  |  | 1 |
| 7911 | Plastic repair of aortic valve |  |  | 1 |
| 7911y00 | Other specified plastic repair of aortic valve |  |  | 1 |
| 7911.11 | Aortic valvuloplasty |  |  | 1 |
| G541400 | Aortic valve stenosis with insufficiency | 1 |  |  |
| 7911411 | Aortic valve repair NEC |  |  | 1 |
| G541100 | Aortic stenosis, non-rheumatic | 1 |  |  |
| G541300 | Aortic stenosis alone, cause unspecified | 1 |  |  |
| G541500 | Aortic stenosis | 1 |  |  |
| G541012 | Aortic regurgitation, non-rheumatic |  | 1 |  |
| G541212 | Aortic regurgitation alone, cause unspecified |  | 1 |  |
| G121.12 | Aortic regurgitation - rheumatic |  | 1 |  |
| G541011 | Aortic insufficiency, non-rheumatic |  | 1 |  |
| G541211 | Aortic insufficiency alone, cause unspecified |  | 1 |  |
| G541000 | Aortic incompetence, non-rheumatic |  | 1 |  |
| G541200 | Aortic incompetence alone, cause unspecified |  | 1 |  |
| G121.11 | Aortic incompetence - rheumatic |  | 1 |  |
| 7911000 | Allograft replacement of aortic valve |  |  | 1 |
| I06.0 | Rheumatic aortic stenosis | 1 |  |  |
| I06.2 | Rheumatic aortic stenosis with insufficiency | 1 |  |  |
| I06.1 | Rheumatic aortic insufficiency |  | 1 |  |
| I35.0 | Nonrheumatic aortic (valve) stenosis | 1 |  |  |
| I35.1 | Nonrheumatic aortic (valve) insufficiency |  | 1 |  |
| I35.2 | Nonrheumatic aortic (valve) stenosis with insufficiency | 1 |  |  |
